# New Approaches to Clinical Trials for Rare Diseases: Decentralized Trial Design for Neurofibromatosis Type 1 and Schwannomatosis

**DOI:** 10.3390/cancers18152463

**Published:** 2026-07-31

**Authors:** Vanessa L. Merker, Shivani Ahlawat, Robert A. Avery, Diana Bradford, Andrea M. Gross, Jennifer Janusz, Andrés J. Lessing, Linda Manth, Miranda L. McManus, Beverly Oberlander, Dominique C. Pichard, William Riter, Kavita Y. Sarin, Steven Sheard, Russell Taylor Sundby, Karin S. Walsh, Pamela L. Wolters, Brigitte C. Widemann, Scott R. Plotkin

**Affiliations:** 1 Department of Neurology and Cancer Center, Massachusetts General Hospital, Boston, MA 02114, USA; 2 The Russell H. Morgan Department of Radiology and Radiological Science, Johns Hopkins University, Baltimore, MD 21205, USA; 3 Division of Ophthalmology, The Children’s Hospital of Philadelphia, Philadelphia, PA 19104, USA; 4 Center for Drug Evaluation and Research, U.S. Food and Drug Administration, Silver Spring, MD 20993, USA; 5 Pediatric Oncology Branch, Center for Cancer Research, National Cancer Institute, National Institutes of Health, Bethesda, MD 20892, USA; 6 Children’s Hospital Colorado, University of Colorado School of Medicine, Aurora, CO 80045, USA; 7 Neurofibromatosis Northeast, Burlington, MA 01803, USA; 8 Department of Biology, College of Charleston, Charleston, SC 29424, USA; 9 Neurofibromatosis Network, Wheaton, IL 60187, USA; 10 Division of Rare Diseases Research Innovation, National Center for Advancing Translational Sciences, Bethesda, MD 20892, USA; 11 Response Evaluation in Neurofibromatosis and Schwannomatosis International Collaboration, Boston, MA 02114, USA; 12 Department of Dermatology, Stanford University School of Medicine, Redwood City, CA 94063, USA; 13 Brainy Ridge, Inc., Bethesda, MD 20817, USA; 14 Children’s National Research Institute & The George Washington School of Medicine & Health Sciences, Washington, DC 20037, USA

**Keywords:** neurofibromatosis, schwannomatosis, clinical trials, United States Food and Drug Administration, telemedicine, digital health

## Abstract

Background: In decentralized clinical trials, some or all activities occur outside of traditional sites, which may reduce time away from school/work and decrease participation burden for patients and their parents/caregivers. This methodology may improve recruitment and retention in studies, which is important for rare diseases like neurofibromatosis type 1 (NF1) and schwannomatosis (SWN). Published guidance exists for the general conduct of decentralized trials, but specific considerations for clinical trial design and endpoints in NF1/SWN have not yet been explored. Methods: The Response Evaluation in Neurofibromatosis and Schwannomatosis (REiNS) International Collaboration is a group of researchers, clinicians, and people affected by NF1 and SWN whose shared goal is to advance clinical trial methodology for NF1/SWN. In December 2023, REiNS members met to discuss the opportunities and challenges of conducting NF1/SWN decentralized trials. Results: Endpoints that are promising for use in NF1/SWN decentralized trials include visual acuity (as tested by the computerized amblyopia treatment study HOTV testing algorithm); electronic versions of REiNS-recommended patient reported outcome measures; digital health technologies for functional outcomes; radiography and computed tomography scans for imaging outcomes; remote photography to assess cutaneous neurofibromas; “e-centralized” evaluations of neurocognitive functioning; and remote biomarkers collected with analyte stabilizing tubes and self-collection devices. Conclusions: Further research is necessary to validate endpoints for decentralized trials for NF1/SWN and evaluate their feasibility. However, trial designs that incorporate decentralized elements hold considerable promise for rare diseases like NF1/SWN where patients encounter significant barriers to traditional clinical trial participation.

## Simple Summary

Many patients with neurofibromatosis type 1 (NF1) and schwannomatosis (SWN) face significant barriers to participating in traditional clinical trials, especially due to travel burden. In this paper, NF1/SWN researchers, clinicians, and patient representatives explore how “decentralized” trials—where some activities take place at home, through telehealth, or with local providers—could make studies more accessible. The authors identify clinical trial measures that are easiest to decentralize and which have challenges that must still be addressed. They highlight promising approaches such as remote symptom assessments, novel digital health tools, at-home biomarker collection, and collaborating with local providers for imaging and eye exams. However, they also emphasize the need to ensure data quality with new measures and potential difficulty navigating legal regulations. Overall, this research methodology could help make clinical trials more patient-friendly and inclusive, leading to broader participation and advancing development of new treatments for NF1/SWN.

## 1. Introduction

Neurofibromatosis type 1 (NF1) and schwannomatosis (SWN) are rare conditions that share a genetic predisposition to peripheral nerve sheath tumors [1]. People with NF1 develop neurofibromas and may have a range of histologically benign and malignant tumors as well as non-tumor manifestations such as cognitive deficits, skeletal abnormalities, and psychosocial challenges. Most manifestations of NF1 begin to be identified in young children [2,3,4] and persist throughout the lifespan [5,6]. People with SWN may develop tumors including schwannomas, meningiomas and ependymomas and non-tumor manifestations such as ocular issues and mononeuropathies. These manifestations typically emerge in adolescence or young adulthood for people with *NF2*-related schwannomatosis; symptomatic presentation in childhood is more likely to be based on ophthalmic or cutaneous features and to occur in people with high-risk genotypes [7,8,9,10]. NF1 and SWN can both cause substantial and progressive morbidity with limited effective treatments currently available.

Over the past two decades, there have been an increasing number of pediatric and adult clinical trials to identify drugs that provide clinical benefit and reduce mortality for individuals with NF1 and SWN [11]. Currently, these trials are conducted almost exclusively at specialized medical centers, which limits access for patients who cannot travel to these institutions. Even for patients where travel is feasible, the burden of participation can be substantial. For this reason, decentralized clinical trials (DCTs), in which some or all trial-related activities occur at locations other than traditional clinical trial sites [12], hold significant promise to increase access to NF1/SWN clinical trials.

While traditional clinical trials take place entirely within hospitals or academic centers, DCTs incorporate activities that occur virtually (e.g., data collection via phone, video, or other technology); in participants’ homes (e.g., by visiting healthcare providers); or within participants’ local communities (e.g., pre-existing care teams, local laboratories, and retail clinics). Fully decentralized trials handle all procedures—from participant enrollment to treatment administration to outcome assessment—outside of traditional trial sites. Alternately, many trials are partially decentralized, with more complex or specialized trial procedures conducted within hospitals and other procedures conducted remotely. In this case, investigators need to select the specific trial components (Table 1) that can be best delivered remotely based on the investigational product, patient population, availability of validated tests, and relevant ethical and privacy concerns [13].

The use of DCTs has grown rapidly with the rise of digital health technologies [14] and pivot from in-person assessments during the COVID-19 pandemic [15]. Clinical trials with decentralized components have led to regulatory approvals from the Food and Drug Administration (FDA) [16,17,18], bolstering the case for their expanded adoption. The FDA, European Medicines Agency (EMA), and other regulatory bodies recently issued guidance on DCTs [12,19,20], and large, international stakeholder groups are working to develop best practices for the implementation of decentralized trial elements [21,22]. While some NF1/SWN clinical trials have already adopted fully or partially decentralized designs [23,24,25], no structured assessment of the benefits, challenges, and opportunities of DCTs in this population has yet been performed.

## 2. Materials and Methods

The REiNS International Collaboration includes researchers, clinicians, and patient representatives who strive to develop meaningful standardized response criteria and novel trial designs for patients with NF1/SWN [26]. REiNS is organized into topical working groups focused on specific outcome domains (e.g., imaging, functional, visual, patient-reported, neurocognitive outcomes), methodologies (e.g., biomarkers), tumor manifestations (e.g., cutaneous neurofibromas), therapy types (e.g., gene therapy), technologies, (e.g., artificial intelligence), and stakeholder groups (e.g., patient representatives). REiNS members across all working groups met on 4 December 2023 in Baltimore, Maryland to discuss opportunities and challenges raised by DCTs. The meeting was attended by 89 participants in-person and online. Approximately 58% were clinicians and researchers across eight specialties, 29% were patient representatives, 11% were representatives from industry, and 2% were representative from regulatory agencies. Patient representatives largely resided in the U.S., and included adults with NF1, *NF2*-related SWN, and other SWN subtypes as well as family members of individuals with NF1 and *NF2*-SWN.

This paper summarizes the research and recommendations presented at the 2023 REiNS Winter Meeting on potential endpoints for DCTs in NF1/SWN. First, we share general considerations for DCTs in NF1/SWN, providing a high-level overview of potential benefits and challenges of DCTs based on published literature, input from the Food and Drug Administration (FDA), and the perspectives of REiNS patient representatives. Second, we summarize the recommendations from seven REiNS working groups regarding which decentralized elements could potentially be incorporated within NF1/SWN clinical trials.

## 3. General Considerations for Decentralized Clinical Trials in NF1/SWN

DCTs offer many potential benefits as well as inherent challenges (Table 2). Fewer in-person visits should decrease the time and financial burden associated with travel to trial sites, opening trial participation over wider geographic areas and to patients with more limited resources. Decreased travel burden may be particularly impactful for children, adolescents, and their caregivers, by minimizing disruptions to children’s school attendance and participation in extracurricular activities, reducing time caregivers must take off from work and other responsibilities to accompany their children to appointments, and lessening potential impacts on siblings and other family members whose schedules must be adjusted to accommodate travel. With improved access and convenience, trials may see faster accrual, greater inclusion of underrepresented groups, and better retention. Furthermore, the use of technology such as smartphones, may improve the engagement and response rates of adolescents and young adults given their preference for digital communication [27]. However, there remains a risk of decreasing access to clinical trials for individuals who do not have or are uncomfortable using smartphones or the internet, potentially excluding older, less educated, or lower income patients. Furthermore, relying on virtual, self-administered assessments may increase patient burden related to data collection and decrease patients’ sense of connection to trial staff and thus engagement in the trial.

Virtual data can be collected more frequently and in more naturalistic settings than traditional clinical outcome assessments, potentially enabling trial endpoints that are relevant to and reflective of patients’ daily functioning (e.g., pain assessments collected daily via e-diaries rather than in-person at clinic visits). However, these outcomes may be more difficult to standardize remotely, and caregivers may need training to appropriately facilitate administration of virtual assessments to young children. These outcomes would also require rigorous validation in this setting, with implementation of procedures to minimize the risk of missing or inaccurate data. Similarly, collecting data using facilities closer to participants (e.g., local imaging or laboratory providers) may be more convenient for participants, but would require rigorous validation to ensure inter-platform variation does not compromise data reproducibility.

Sponsors of DCTs may benefit from reduced site activation and monitoring costs as a single site can remotely enroll a larger number of patients. However, the initial start-up costs for trials may be greater, especially when providing and validating digital health technologies. Given the costs and risks associated with trialing new designs and endpoints, sponsors may be hesitant to adopt decentralized elements without proven regulatory acceptance. Sponsors and investigators must also navigate several evolving legal and regulatory issues. Investigators must clearly distinguish between trial activities considered as research and those considered as clinical provision of telehealth, since research interventions are governed by institutional review boards while telehealth is governed by state and federal laws regarding licensure. For example, remotely collecting toxicity data during a study can be considered research, but if a participant requires medical decision making to address the toxicity (e.g., change in dose or prescription of a new medication), this could be considered medical care. Another complex issue for decentralized medical trials is the delivery of investigational agents across state lines for patients that do not travel to trial sites [28]. Given the complex web of institutional, state, and country-specific regulations that govern clinical trial conduct, sponsors and investigators must carefully consider the specific locations and tasks involved in each decentralized trial to maintain compliance.

### 3.1. FDA Guidance on Decentralized Trials

FDA’s guidance for industry, investigators and other stakeholders on DCTs was released in 2023 and finalized in 2024 [12]. The guidance provides recommendations and considerations for the conduct of DCTs, but importantly, regulatory requirements for investigation of drug products are the same regardless of whether decentralized elements are employed.

FDA’s guidance describes considerations for trial design, remote visits, use of digital health technologies, electronic informed consent, packaging and shipping of investigational products, software used in DCTs, and safety monitoring plans, as well as the roles and responsibilities of sponsors and investigators. Notably, additional planning and pre-specification of key personnel roles and operating procedures may be needed during development of DCTs. Trial-related activities may be delegated to local healthcare providers if they are qualified to perform these activities in their clinical practice and activities do not require detailed knowledge of the protocol or investigator’s brochure (e.g., obtaining vital signs, performing routine physical exams, etc.) While investigators of drug trials subject to 21CFR312.53 are required to complete an FDA Form 1572 to describe their qualifications and commitment to following legal regulations, local healthcare providers who perform trial-related activities in a DCT do not require a Form 1572; however, sponsors must plan for how trial-related data gathered from these providers will be documented. Data quality safeguards should be used to limit variability in data collection across individuals/sites, such as using detailed protocols, standard operating procedures, and training modules, and/or video supervision via telehealth. FDA’s guidance describes considerations for data management plans and safety monitoring plans that account for decentralized elements and multiple sources of data. Examples include describing how both trial participants and local healthcare providers will report any adverse events, and detailing how and where participants should seek medical assistance if needed (e.g., through local emergency services, tele-triage with central staff, etc.).

Decentralized elements may decrease patient burden and improve recruitment of a diverse clinical trial population, thus resulting in better representation of the population in which a product is intended for use. However, as discussed in the FDA guidance, the decision to implement decentralized elements should consider the specific product, study population, stage of development, and safety profile. Investigators must also ensure that use of decentralized trial elements complies with guidance from other relevant agencies, including the Office for Human Research Protection (OHRP), which may have additional regulations. For example, OHRP and/or local institutional review boards may consider remote healthcare staff as ‘engaged in research’ even when they are not classified as sub-investigators per FDA guidance.

### 3.2. NF1/SWN Patient Perspectives on Decentralized Trials

Members of the REiNS patient representative working group, which includes adults with all types of NF1 and SWN as well as the family members/caregivers of children with NF1 and SWN from multiple countries [29], were enthusiastic about DCTs, citing the convenience of being able to participate from their home or local clinic. With many rare disease patients living hundreds of miles from trial sites, decentralized designs could afford more people with NF1/SWN the opportunity to participate in clinical trials. Reducing trial-related travel and associated absences from school, work, and extracurricular activities may particularly benefit pediatric and young adult participants by helping safeguard the social and academic development of a population already prone to challenges in these areas [30,31,32]. In addition to the many potential benefits related to decreasing the burdens of trial participation, decentralization may help patients less familiar with clinical research feel more comfortable participating because they can receive trial-related assessments from their pre-existing local providers. Decentralization may also make trials more representative by increasing participation of patients historically underrepresented in clinical trials [33].

REiNS patient representatives also expressed concerns regarding decentralization that must be considered moving forward. DCTs often require use of technology, yet patients may not have access to high-speed internet or needed devices, may lack technical skills, and may have security and privacy concerns with remote data collection. Coordination with local healthcare providers could be challenging; they may not have necessary resources, including time and staff, to participate in DCTs and may not be invested in clinical trials, putting the onus on the patient to encourage their local provider’s involvement. In addition, one of the benefits patients identify to participating in a traditional clinical trial is the personal relationship with the site principal investigator, who may be the only NF1/SWN expert to which they have access; this relationship may not be as strong in DCTs with less in-person interaction. Furthermore, patient representatives in the United States noted a large concern related to the cost of trial participation, even in DCTs that might require less travel but could still incur co-pays or fees for local services. European patient representatives also noted challenges with DCTs crossing national borders, including obstacles posed by linguistic and cultural differences and strict data privacy laws that can pose problems with data transfer.

To overcome these challenges, patient representatives stressed that communication between the study team, patients, and local providers must be well-established and clear. Patient representatives also proposed that DCTs offer incentives to encourage patient and provider retention and/or retain a dedicated patient navigator to help patients with trial logistics and insurance approvals. A joint tumor board or other clinical support from trial staff could also help engage local providers and support their continued participation in DCTs.

## 4. Working Group Recommendations on Decentralized Elements for NF1/SWN Clinical Trials

Seven REiNS working groups identified potential decentralized evaluations and outcomes of interest for NF1/SWN clinical trials, which they collaboratively discussed within regular working group meetings and presented at the 2023 Winter Meeting for wider feedback. Based on this feedback, meeting presenters and working group leaders refined the recommendations presented in this manuscript as a starting point for expanding decentralized trial elements in NF1/SWN. However, no formal voting was conducted to ratify recommendations across working groups and as such, individual members may hold a range of viewpoints about this topic. In the sections below, each working group identifies relevant techniques and measures that could potentially be decentralized, outlines the key benefits and challenges to decentralizing these trial elements, and assesses which elements should be prioritized for testing and validation within NF1/SWN DCTs based on the expert opinion of REiNS members (Table 3).

### 4.1. Functional Outcomes

People with chronic conditions such as NF1/SWN often experience disability and morbidity, and being able to reliably evaluate whether interventions improve symptoms or function is a key goal of clinical trials. The REiNS Collaboration has recommended functional evaluations to address specific aspects of NF1/SWN including hearing, facial function, pulmonary function, sleep and strength [34,35,36]. To date, all recommended primary and secondary trial endpoints have been evaluated in a centralized manner at research institutions. 

Recent advances in technology, including the broad availability of smartphones and mobile applications, raise the possibility that some functional evaluations could be completed from home, which could improve accessibility and ease of participation in clinical trials. For example, for those with *NF2*-SWN associated hearing loss and/or facial nerve dysfunction, there are multiple currently available mobile applications for measuring hearing at home and measuring facial function in people with nerve palsy [37,38,39]. Wearable devices that could monitor physical functioning and sleep also provide an intriguing possibility for expanding naturalistic outcomes of activity. However, choosing clinically meaningful, valid, and reliable trial endpoints derived from these devices will require significant input from experts and the patient community.

Another option for DCTs is better integrating local evaluations that fall within standard clinical practice of local providers. For example, the recommended audiology testing for people with *NF2*-related SWN includes evaluation of word recognition score and pure tone thresholds. Both are standard practice assessments that could be performed by a local medical provider rather than at a central study location that may be a long distance from the participant’s home, thus decreasing the burden of trial participation. The key challenge, however, will be ensuring that locally performed functional evaluations are done in a consistent manner with reliable results.

### 4.2. Visual Outcomes

Visual acuity (VA) is the primary functional measure used to assess treatment efficacy for children with optic pathway gliomas associated with NF1 (NF1-OPG). VA is the clarity of vision, as determined by the ability to differentiate optotypes at a distance. Children are typically between 1 and 7 years old when their NF1-OPG is discovered, requiring VA testing that can accommodate children with varying developmental and cognitive skills. While Teller Acuity Cards is recommended as a primary outcome measure for NF1-OPG clinical trials [40], its cost and the expertise needed for administration make it infeasible for DCTs. However, the computerized amblyopia treatment study HOTV VA testing protocol is currently used as a secondary outcome in clinical trials, for children who are typically older, more cooperative and know some of their letters [41]. This protocol can be run by any eye care professional using a software program that requires minimal training—making it ideal for DCTs.

Testing the visual field and the presence/absence of optic atrophy are both relevant and important outcomes [42], but the expertise necessary for the former and the subjective nature of the latter make both impractical for DCTs. However, as optical coherence tomography becomes more ubiquitous and demonstrates success as a relevant biomarker [43], it could potentially be included in DCTs, even with known differences between commercial devices.

Finally, there is an opportunity to determine trial eligibility and monitor treatment-related toxicity remotely in DCTs, as most exclusion criteria can be readily identified by local providers and thus far, treatment-altering ocular toxicity in children with NF1-OPG has been exceedingly rare.

### 4.3. Imaging Outcomes

Incorporating imaging outcomes into DCTs requires careful consideration of imaging acquisition and interpretation. Successful multicenter trials need rigorous standardization of imaging platforms and protocols, with precision testing of the modality and post-processing methods to ensure data reproducibility across sites. While this is feasible for modalities such as radiography and computed tomography, it is more challenging for magnetic resonance imaging (MRI)—especially when study imaging protocols deviate from typical clinical care, increasing the risk of technical failure [44]. As such, not all modalities may be equally suitable for decentralization. For DCTs requiring advanced imaging such as MRI, study participants should undergo imaging under similar conditions (same coils) using the same platform (magnet strength) and imaging protocol (field of view and sequence parameters) for longitudinal assessment.

Imaging interpretation can range from fully decentralized (local imaging reads incorporated as trial outcomes) to fully centralized (imaging uploaded to a central repository and interpreted by trained trial staff) or hybrid (local interpretation combined with central review). Fully centralized or hybrid imaging interpretation may appeal to study participants by providing access to expertise in NF1/SWN otherwise unavailable locally. Among the various manifestations of NF1 and SWN, intracranial neoplasms (such as glioma and vestibular schwannoma) and musculoskeletal sequalae of NF1 (e.g., scoliosis, low bone mineral density, long bone dysplasia, tibial pseudoarthrosis) may be more suitable for DCTs based on greater clinical trial experience [45,46,47,48,49,50], established clinical imaging guidelines [51,52], and/or use of more straightforward imaging strategies available at most radiology practices.

### 4.4. Patient-Reported Outcomes

Patient-reported outcomes measures (PROMs) document clinical benefit in trials and are supported by the FDA and EMA for regulatory approval and labeling claims [53,54]. Remote electronic PROMs (ePROMs) have proven feasible in both medical [16] and psychosocial [23] clinical trials, with several advantages: ePROMS (1) are largely equivalent to paper forms [55] and preferred by the FDA [56], EMA [57], and most patients [58]; (2) collect more accurate and complete data compared to paper [58,59]; (3) assess real-time symptoms that may provide more valid results [60]; and (4) allow for the option for participants to use their personal devices for data collection (termed Bring Your Own Device; BYOD) [61].

However, challenges remain regarding remote ePROM administration in clinical trials. Few trials using BYOD have supported product approval and labeling claims to date [62], and specifics of ePROM administration are not well-described in labeling or publications, making it difficult to track the use of BYOD in trials. Reliability and validity may be affected when adapting paper PROMs to electronic format and when administering ePROMs remotely, especially for children and patients with poor literacy. Some patients report concerns about data security and privacy [61,63]. Finally, implementing ePROMs requires funding to support digital technology.

To address these challenges, user-friendly mobile and web-based platforms are available for ePROMs that do not require personal identifiable information, and both BYOD and study-provided devices can be used in DCTs. Current REiNS-recommended PROMs are available electronically, and other paper measures can be adapted following best practices [64]. Strategies to facilitate remote ePROMs include collaborating with patient representatives to develop ePROM systems, training staff and patients in ePROM technology [65], maintaining ongoing communication with DCT participants for technical support, sending automated reminders, and offering text-to-speech to facilitate comprehension and accessibility, especially for children and patients with reading difficulties. Digital systems could be funded through grants, foundations, or pharmaceutical companies to enable ePROM data collection for NF1/SWN clinical trials.

### 4.5. Cutaneous Neurofibroma Outcomes

Cutaneous neurofibromas (cNFs) are among the most common and visible manifestations of NF1 and can cause pain, pruritus and substantial psychosocial burden [66]. Because cNFs are often numerous and distributed across multiple body sites, scalable and reliable outcome measures are needed for clinical trials. REiNS has previously emphasized the need for standardized cNF assessment methods [67]; however, validated clinical trial endpoints for cNF burden remain limited.

Some cNF trial elements are readily amenable to decentralization, including recruitment, electronic patient-reported outcome measures, study medication administration, adherence monitoring, and safety assessments [13]. Cutaneous toxicity monitoring may be particularly feasible for remote assessment because many dermatologic adverse events can be graded using visible skin features [68]. In contrast, decentralizing cNF efficacy assessments is more challenging. Potential approaches include measurement by local providers, participant- or caregiver-assisted measurement guided by telemedicine, standardized patient-submitted photographs, and local imaging. These approaches could reduce travel burden, expand participation beyond specialized NF centers, and allow more frequent longitudinal assessment of visible or symptomatic lesions.

However, cNF assessment presents several challenges that must be addressed before decentralized efficacy endpoints can be broadly implemented. cNFs are three-dimensional lesions with ill-defined borders and substantial variation in color, morphology, size, subtype, and anatomic distribution. Remote or local assessments introduce additional sources of variability, including differences in lighting, camera distance, body positioning, image quality, lesion selection, measurement technique, and local provider experience with NF1. Researchers should address these challenges by developing standardized photography and measurement protocols, calibrated imaging methods, central review, and prospective comparison with centralized reference standards.

The highest priority for cNF decentralized trials is to determine which remote or local measures are reliable enough for endpoint use. Cutaneous toxicity monitoring using photography and telemedicine could be an accessible option for NF1/SWN decentralized trials as it has already been used in other skin disorders, although this would require feasibility testing in this disease and setting [69]. In contrast, decentralized cNF measurement remains important but requires further validation before use as an efficacy endpoint [70]. Priority studies should assess test–retest reliability, inter-rater agreement, sensitivity to change, feasibility across diverse skin tones and body sites, and concordance with centralized expert assessment. These validation studies will be essential before decentralized cNF measures can support efficacy conclusions in interventional trials.

### 4.6. Neurocognitive Outcomes

When considering decentralizing cognitive assessments for clinical trials, potential options include (1) a completely decentralized approach in which community psychologists perform study-specific neurocognitive assessments and transfer data to a coordinating center, and (2) an “e-centralized” approach with a centralized team evaluating individuals virtually at home or at their local doctor’s office. In response to the COVID-19 pandemic, many publishers developed cognitive test versions that could be used remotely, and the use of virtual platforms has become routine for many researchers, clinicians, patients and their families.

While there are potential benefits to DCTs, neurocognitive assessment is a specialized skill that requires specifically trained psychologists who typically conduct in-person evaluations. Virtual assessment is still relatively new, raising concerns surrounding validity and reliability. For a fully decentralized approach, multiple considerations arise: (1) limited availability of local psychologists to conduct study evaluations, (2) limited access to research-specific test instruments for community psychologists, (3) financial support for community psychologists willing to engage in research assessments, and (4) ensuring data validity, integrity, and standardization. Regarding “e-centralized” possibilities, there are unique considerations including (1) limitations in what can be assessed virtually; (2) limited number of tests that are currently validated for virtual use, (3) limits to what age range can be assessed, expecting that very young children may not be reliably evaluated in this format; and (4) ensuring an appropriate environment for virtual assessment (e.g., verifying there are no devices that may enhance or distract from performance; educating parents on test processes and potentially involving them as partners in the evaluation) which may require additional preparatory appointments. Ultimately, decentralizing or “e-centralizing” cognitive assessment within clinical trials will require additional administrative planning and support and subsequently greater financial support for such trials.

Given interest from patients in novel trial methods and the need to address inequities in access to research, neuropsychologists involved in NF1/SWN clinical trials are encouraged to conduct prospective studies establishing the validity and reliability of virtual cognitive evaluation in this population. Given the challenges with fully decentralized cognitive studies, it is likely that the field will need to develop “e-centralized” approaches moving forward.

### 4.7. Biomarker Studies

Decentralizing biomarker collection in clinical trials holds tremendous promise for decreasing the patient burden of participation. Empowering local or home collection of correlatives may increase compliance and feasibility for serial timepoint collections which have been shown to improve accuracy in NF1 cell free DNA (cfDNA) assays [71,72] and have been recommended by REiNS for biospecimen collection in all trials involving surgical interventions [73]. Furthermore, decreased travel requirements may increase enrollment of resource-limited and geographically isolated populations, thereby increasing study power. Decentralized biomarker studies can even be structured as patient-initiated or patient-partnered trials, which have catalyzed genomic discoveries in other rare diseases using the Count Me In platform [74].

Recent technological advances have made blood-based biomarker collection for decentralized trials more feasible. Analyte stabilizing tubes, which stabilize nucleic acids for up to 14 days, enable room temperature storage and transport of samples from a remote site to a central processing core [75]. Dried blood spot cards, used for remote diagnostics for over a century [76], have recently been used to detect cfDNA in cancer patients, demonstrating similar fragment profiles to those observed in plasma cfDNA [77]. Finally, novel devices enable self-collection of capillary blood, potentially negating the need for a local phlebotomist in DCTs. Devices like Tasso+ (Tasso, Inc., Seattle, WA, USA) and TAP II (YourBioHealth, Medford, MA, USA) have shown that 80% of participants can successfully collect blood on their first attempt, and 98.6% can do so within two attempts [78]. The concordance between capillary and venous samples is high, ranging from 94.7% to 99.5% and many participants prefer self-collection over venous blood draws [78]. Self-collection has been successfully used to collect and analyze multiple analytes, including RNA [79].

Technological advances have also increased feasibility of decentralizing tissue-based biomarker correlatives. Digital pathology enables high-resolution sharing of images for central review. Recently, studies have used deep learning with digital whole slide images to predict clinically relevant molecular subtypes and survival in rhabdomyosarcoma [80].

However, implementation of decentralized biomarkers comes with challenges. Institutional costs may rise with the use of analyte stabilizing tubes, novel devices for collection, and/or third-party phlebotomy and sample storage. Additionally, decentralized exploratory correlatives may not always be paired with “ground truth” standard diagnostic methods, e.g., imaging. Finally, the interpretation of exploratory biomarkers may be complicated when relying on patient-reported outcomes for interpretation of results or on evaluations from local providers with limited experience in NF1/SWN who may use outdated terminology or guidelines.

Solutions to these challenges include developing guidelines for uniform collection times and annotation to ensure reliable biomarker data [73,81]. Histology and AI-assisted digital pathology platforms can enhance diagnostic accuracy through centralized review [80,82,83,84]. Furthermore, as DCTs become more common, the costs associated with sample collection, processing, and storage are expected to decrease.

## 5. Conclusions

In summary, the REiNS International Collaboration 2023 Winter Meeting identified exciting opportunities to expand access to NF1/SWN clinical trials using decentralized methodologies. Discussion amongst diverse stakeholders identified several potential benefits of decentralization particularly relevant for this community, including decreasing patients’ travel burden to the few specialized centers in NF1/SWN that traditionally host clinical trials, potentially enhancing recruitment and retention of diverse participants, and expanding opportunities for new virtually collected endpoints that may better reflect patients’ day-to-day functioning. Particularly for children, adolescents, and young adults with NF1/SWN, decentralization may reduce the disruption caused by traditional trial participation and expand access to investigational treatments. As such, the additional efforts needed to implement decentralized elements—such as navigating regulatory guidance across agencies and creating processes to standardize remote data collection—are worthwhile endeavors to improve NF1/SWN clinical trials. To quote a recent National Cancer Policy Forum meeting report, “The question is no longer whether we can conduct aspects of … trials in a decentralized fashion, but how we can best prioritize the most useful DCT methods moving forward, and prospectively design DCT studies that maintain patient safety and data integrity.” [85].

**Figure 1:**
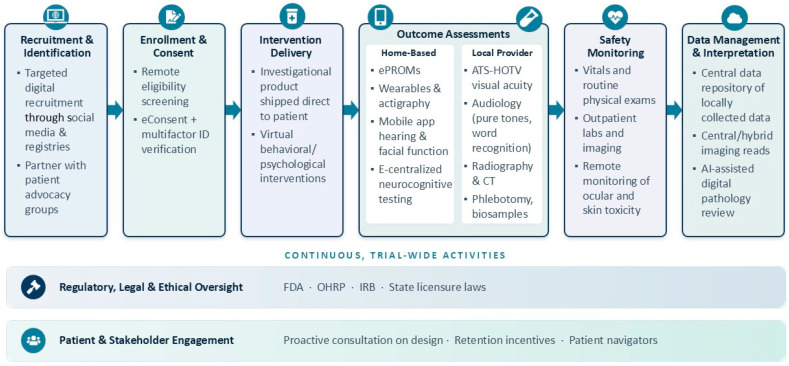
Potential Decentralized Elements for NF1/SWN Clinical Trials.

To this end, REiNS recommends investigators begin implementing some remote outcomes to formally assess the feasibility of decentralized elements in NF1/SWN clinical trials (Figure 1). Currently, the most promising options for virtual collection within patients’ homes include electronic patient-reported outcome measures to assess pain, quality of life, and other domains; validated e-centralized neurocognitive tests; actigraphy and other functional outcomes assessed using wearable devices; and monitoring of cutaneous toxicity using photographs and telehealth modalities. The most promising measures for decentralized use within local medical providers’ offices and laboratories include ATS-HOTV testing of vision; word recognition and pure-tone averages to assess hearing; and radiography and/or CT scanning to assess musculoskeletal issues. 

However, additional research is needed to validate these and other decentralized endpoints, with a crucial focus on demonstrating the reliability, accuracy, and reproducibility of remotely collected data to ensure the continued generation of robust clinical trial data. Implementing decentralized outcomes will also require: (1) standardized protocols, operating procedures, and trainings to minimize variability in data collection; (2) data management plans to specify how remote data will be captured, transmitted, stored, and interpreted; and (3) provision, validation, and technical support for any digital health technology required for outcome assessment. Researchers and product developers should proactively engage regulators during each of these tasks to ensure acceptance of new trial designs and endpoints during regulatory decision-making. Moving forward, the NF1/SWN community should also continue to facilitate patient and stakeholder engagement in decentralized trial design and implementation to maximize potential benefits to all parties and proactively mitigate identified challenges.

## Figures and Tables

**Table 1:** Traditional and Decentralized Trial Components.

Trial Component	Traditional Site-Based	Virtual/Remote Options
Recruitment	Hospital-based recruitment	Targeted digital recruitment
Enrollment	In-person consent	eConsent and multifactor identity verification
Product Distribution/ Intervention Delivery	Medications dispensed and reconciled by study staff at in-person appointments In-person psychological, cognitive, and other interventions	Medications may be shipped directly to patient Psychological, cognitive, and other interventions delivered virtually
Safety Assessment	Hospital-based assessments	Monitoring at local outpatient facilities, labs, and imaging centers Digital platforms for adverse event self-reporting
Outcomes Assessment	In-person clinical outcome assessments In-person paper or electronic PROMs/diaries	Electronic patient-reported outcome measures via web/mobile app Assessment at local providers or e-centralized assessment Remote monitoring via wearables and connected health devices

**Table 2:** Potential Benefits and Challenges of Decentralized Trial Implementation.

	Potential Benefits	Potential Challenges
Patient Access to Trials	More equitable geographic and economic access	Excluding people without computers/tablets/smartphones/high speed internet or who are less digitally literate
Patient Convenience	Fewer and/or shorter in-person visits	Increased responsibility for self-assessments
Patient Accrual and Retention	Improved access = faster accrual Decreased burden = better retention	Ensuring patient engagement in a virtual world
Data Collection	More frequent and real-time data collection possible	Risk of poor quality data; difficulties with data reproducibility and inter-platform variability
Endpoint Selection	New endpoints with more real-world relevance	Need to validate new endpoints; uncertain regulatory status
Costs	Reduced costs for site activation/monitoring	Increased costs for digital health technology

**Table 3:** Outcomes of interest for use in decentralized trials of NF1 and SWN.

Working Group	Outcomes Ready for Testing for Decentralized Use
Functional outcomes	Hearing outcomes Actigraphy
Visual outcomes	ATS-HOTV
Imaging outcomes	Radiography CT
cNF outcomes	Cutaneous toxicity
Patient-reported outcomes	ePROMs
Neurocognitive outcomes	Validated e-centralized neurocognitive tests
Biomarkers	Blood samples collected through analyte stabilizing tubes, blood spot cards, or self-collection devices

Abbreviations: ATS-HOTV, amblyopia treatment study HOTV; cNF, cutaneous neurofibromas; CT, computed tomography; ePROMs, electronic patient-reported outcome measures.

## Data Availability

No new data were created or analyzed in this study. Data sharing is not applicable to this article.

## Abbreviations

The following abbreviations are using in this manuscript:

BYOD	Bring your own device
cfDNA	cell-free DNA
cNF	cutaneous neurofibroma
DCT	Decentralized clinical trial
EMA	European Medicines Agency
ePROM	electronic patient-reported outcome measure
FDA	Food and Drug Administration
NF1	Neurofibromatosis type 1
NF1-OPG	NF1-associated optic pathway gliomas
MRI	Magnetic Resonance Imaging
PROM	Patient-reported outcome measures
REiNS	Response Evaluation in Neurofibromatosis and Schwannomatosis
SWN	Schwannomatosis
VA	Visual acuity
